# Impact of mobile payment on physical health: Evidence from the 2017 China household finance survey

**DOI:** 10.3389/fpubh.2022.963234

**Published:** 2022-08-01

**Authors:** Rui Zhang, Yunzhi Zhang, Jiahui Xia

**Affiliations:** ^1^Department of Economics, Jinan University, Guangzhou, China; ^2^Faculty of Law, Economic, and Management, LEO-University of Orléans, Orléans, France; ^3^School of Management, Jinan University, Guangzhou, China

**Keywords:** mobile payment, physical health, commercial insurance, leisure consumption, heterogeneity analysis, mechanism

## Abstract

Individuals' health status is an essential indicator of the overall strength of a country. Existing literature has studied the determinants of individuals' health, but there is no direct evidence to date on the impact of mobile payment on health. To supplement relevant research and provide insightful policy suggestions to families, government and societies, based on data of 32,058 observations from the 2017 China Household Finance Survey, we estimate the effects of mobile payment on physical health using ordinary least squares and two-stage least squares strategy. This paper provides direct evidence that mobile payment has a positive impact on citizens' physical health. Heterogeneity analysis shows that mobile payment has a more profound impact on the health of citizens who are rural and less educated. Finally, further findings in this paper suggest that commercial insurance and leisure consumption are the mechanism through which the link between mobile payment and individuals' health operates.

## Introduction

Citizens' health is an important issue in both developed and developing countries. Individuals' health status not only affects their happiness, labor supply, wages, and productivity, but also family income, asset allocation, and economic well-being (1–5). Therefore, research on the determinants of health has long been a focus of attention in industry and academia. From a macro perspective, urbanization (6), environmental pollution (7), and international trade (8) may affect the health of a country's residents. In addition, other factors, such as the financial crisis (9), minimum wage (10), skilled migration (11), the level of national corruption (12), and extreme weather (13) may also exert an impact on their health. From a micro perspective, personal religious beliefs (14), social capital (15, 16), self-employment (17), physical exercise (18), unemployment (19), and income (20) can affect residents' health.

In recent decades, the emergence of the internet and the digital economy has changed human lifestyles and social behaviors. These changes may have an impact on people's health. Against this background, research on the determinants of health should focus not only on traditional perspectives but should also take internet use into account (21, 22). The development of the internet, which has enabled mobile payment, online shopping, and online ordering, makes daily life more convenient and information more accessible. In particular, mobile payment has been incorporated into people's way of life and has taken off in recent years. On the one hand, mobile payment has facilitated private consumption, while on the other it has enabled better access to social services, provident funds, medical services, transportation and other government services. Generally speaking, mobile payment is gradually replacing traditional cash payment methods and becoming an important way of carrying out transactions.

To date, there has not yet been an empirical study that examines the direct link between mobile payment and people's physical health. An urgent need for academic evidence on the question of whether mobile payment has an impact on health is raised. As the abovementioned studies indicate, mobile payment could have an impact on individuals' health through several channels. First, the use of mobile payment smooths consumption, which helps improve the diversity of food types in the family's daily diet and thus ensures that the individual's nutritional intake is more balanced, ultimately affecting the individual's health. Second, the use of mobile payment reduces transaction costs and facilitates access to online private insurance and medical services, which in turn affects the individual's health. To fill the gap in the literature on the direct link between mobile payment and individuals' health, we investigate the impact of mobile payment on residents' physical health in the case of China, using a dataset sourced from the 2017 China Household Finance Survey.

This paper focuses on China for two reasons. First, China has become the largest market in the world for mobile payment, and so it provides the best experimental sample to conduct this research. According to the “Statistical Report on China's Internet Development Status” Released by the China Internet Network Information Center, by the end of 2020, there were more than 850 million users of mobile payment in China, and the proportion of internet users using mobile payment was close to 90%. Mobile payment has brought profound impact to both society and individuals. Against the backdrop of the growing global digital economy, studying the impact of mobile payment on physical health for the case of China is of major significance.

Second, Chinese citizens prefer to go to public hospitals at their country or city to seek medical treatment, and these hospitals usually ask for a deposit before treatment. The use of mobile payment can help patients to quickly obtain loans through certain payment platforms such as WeChat and Alipay, thereby enabling patients to obtain essential medical resources in a timely fashion. Moreover, mobile payment platforms in China such as Alipay and WeChat provide convenient channels for users to purchase various private medical insurance. As an important supplement to public medical services, private insurance can substantially alleviate the financial pressure of treatment, further improve the level of medical services received during treatment, and thereby improve the health of residents.

The contributions of this paper are mainly 4-fold. First, to the best of our knowledge, this is the first paper to provide direct evidence that mobile payment could have an impact on individuals' physical health. Second, this paper enriches the related research on the digital economy by focusing on the effect of mobile payment at the micro level. Third, this paper provides evidence on the mechanisms through which mobile payment affects individuals' physical health. Finally, the results of this paper enlighten families, society, and the government by providing several policy implications against the background of COVID-19.

The remainder of this paper is structured as follows. Section 2 Data and methods describes the data source, and explains the variables and methods. The empirical results are detailed in Section 3 Empirical analysis and results. Further tests such as mechanism analyses are presented in Section 4 Mechanism. Section 5 Discussion discusss the results. Section 6 Conclusions and policy implications provides the conclusions and policy implications.

## Data and methods

### Data sources

The data used in this article are drawn from the 2017 China Household Finance Survey, which uses the Probability Proportional to Size (PPS) sampling method to cover 29 provinces, including Beijing, Guangdong, Wuhan, and Sichuan, and 355 counties (districts and county-level cities), with a sample size of 40,011 households. The overall data are of high quality and are nationally representative. The survey includes not only individual-level personal information such as physical health, marital status, education, age, and gender, but also family-level information such as household size, transfer payments, insurance, and security. The total number of individuals surveyed in 2017 was approximately 127,012. After deleting variables with missing values and outliers in health status, mobile payment usage, etc., we left with 32,058 valid survey responses.

### Variable definition

#### The measurement of physical health

For the measurement of physical health, most studies use respondents' self-evaluation of their health (23–27), and this study does the same. The related question in the questionnaire is “How do you feel about your physical condition?” Respondents can select one of five options: “not good,” “very bad,” “fair,” “good,” and “very good,” corresponding to a score of 1–5, respectively. In our setting, the higher the score, the better the respondents' health status.

#### Measurement of mobile payment

The question about mobile payment in the questionnaire is “Which of the following payment methods do you and your family generally use while shopping (including online shopping)? (Multiple choices available).” Respondents can choose from the following: (1). Cash, (2). Credit card, (3). Payment via computer (Including online banking, Alipay, etc.), (4). Payment via mobile phone, tablet, and other mobile terminals (Including Alipay APP, WeChat Pay, mobile banking, Apple pay, etc.). If the respondent's answer includes option 4, the mobile payment variable is assigned a value of 1, otherwise it is assigned a value of 0.

#### Control variables

In order to alleviate the problem of endogeneity caused by the omitted variables, this paper includes a series of control variables; namely, gender, age, marital status, rural household registration, family size, employment status, home ownership, the family's transfer income, the economic development status of the province, and geographical factors that do not change over time, such as whether the respondent lives in the eastern, central, or western region. From the descriptive statistics shown in Table 1, it can be seen that the mean value of mobile payment is 0.339, meaning just under 34% of people use mobile payment. In addition, the mean of gender is 0.496, indicating that there is not much difference between the numbers of males and females in the sample. The mean of education is 10, which indicate that the education level of residents in China is not high enough. The mean value of agriculture is 0.235, meaning urban residents account for a large proportion in CFPS. The minimum and maximum of variables in Table 1 indicate that outlier is not a concern in our case.

**Table 1:** Descriptive statistics.

**Variable**	**Definition**	**Mean**	**SD**	**Min**	**Max**
Health	Individual's physical health	3.574	0.991	1	5
Mobile payment	Dummy variable equals 1 if the individual uses mobile payment, and 0 otherwise	0.339	0.473	0	1
Gender	1 for male, 0 for female	0.496	0.500	0	1
Age	Individual's age	48.09	17.87	16	117
Education	Years of education	10.00	4.475	0	22
Marital status	Dummy variable equals 1 if the individual is married, and 0 otherwise	0.769	0.421	0	1
Agriculture	Dummy variable equals 1 if the individual is registered in a rural prefecture, and 0 otherwise	0.235	0.424	0	1
Family size	The number of members in the family	2.219	1.481	0	12
Employment status	Dummy variable equals 1 if the individual has a job, and 0 otherwise	0.555	0.497	0	1
Home owner	Dummy variable equals 1 if the individual owns a home, and 0 otherwise	0.808	0.394	0	1
Transfer income	Annual transfer income of household (log)	3.338	3.875	0	13.46
GDP per capita	Annual GDP per capita at province level (log)	11.07	0.395	10.26	11.77
West	Dummy variable equals 1 if household is in western region of China, and 0 otherwise	0.260	0.439	0	1
Center	Dummy variable equals 1 if household is in central region of China, and 0 otherwise	0.577	0.494	0	1
East	Dummy variable equals 1 if household is in eastern region of China, and 0 otherwise	0.163	0.369	0	1

### Methodology

First, to investigate the impact of mobile payment on health, we employ a multiple linear regression model, specified as follows:


(1) $$\begin{array}{l} \text{Healt}h_{i}=\alpha +\beta \text{paymen}t_{i}+\lambda X_{i}+\varepsilon_{i} \end{array}$$

where, *Health*_*i*_ denotes the health status of individual *i;* *payment*_*i*_ is a dummy variable that takes the value 1 if individual i uses mobile payment, and 0 otherwise. X is a vector of the following observable determinants of health status: gender, age, marital status, family size, a dummy for employment status, home ownership, and household transfer income. We also control for the level of economic development of the province and regional dummies for the West, Center and East. ε_*i*_ denotes the error term.

## Empirical analysis and results

### Baseline results

When analyzing the causal relationship between mobile payment and physical health, strong correlations between some explanatory variables may cause multicollinearity problems. Severe multicollinearity leads to unstable results, with a biased estimation of the causal relationship between mobile payment and health. Hence, a test must be carried out to check for multicollinearity. The results in Table 2 show that the variance inflation factor (VIF) of each variable is <3, indicating that multicollinearity is not a concern in our case (28).

**Table 2:** Results of the multicollinearity test.

**Variable**	**VIF**	**1/VIF**
East	2.290	0.437
GDP per capita	1.860	0.536
Age	1.800	0.554
Education	1.520	0.659
Center	1.390	0.721
Employment status	1.290	0.772
Agriculture	1.280	0.779
Mobile payment	1.260	0.796
Marital status	1.240	0.803
Family size	1.170	0.857
Home owner	1.100	0.909
Gender	1.050	0.949
Transfer income	1.030	0.973
Mean VIF	1.410	

Table 3 reports the results of the impact of mobile payment on health. Column (1) includes only the dummy of mobile payment, and the result is positive and statistically significant at the 1% level. We include the control variables in columns (2) and (3) without controlling for and controlling for regional fixed effects, respectively. These results show that the estimation coefficient of mobile payment is still positive and significant at the 1% level. In order to alleviate the impact of heteroskedasticity on the estimation results, column (4) reports the estimation results of clustering at the household level. The relationship between mobile payment and physical health remains statistically significant. Overall, these results indicate that mobile payment has a positive impact on health status. Our preferred estimator is in column (4), indicating that an individual who uses mobile payment has a predicted health score about 0.13 points higher than a comparable individual who does not use mobile payment. Additionally, the effects of gender, education, marital status, family size, employment status, agricultural status, home owner and economic development level have the expected signs and are statistically significant overall in our results. Specifically, gender, education, family size, employment status, home owner and economic development level have positive impact on health status. The estimated coefficients of age and agriculture are negative and significant at the 1% level, indicating that age and agriculture have a negative impact on physical health.

**Table 3:** Results of the impact of mobile payment on health.

**Variable**	**Health**	**Health**	**Health**	**Health**
Mobile payment	0.465***	0.134***	0.130***	0.130***
	(0.011)	(0.012)	(0.012)	(0.015)
Gender		0.028***	0.028***	0.028***
		(0.010)	(0.010)	(0.008)
Age		−0.013***	−0.013***	−0.013***
		(0.000)	(0.000)	(0.000)
Education		0.031***	0.031***	0.031***
		(0.001)	(0.001)	(0.002)
Marital status		−0.016	−0.016	−0.016
		(0.013)	(0.013)	(0.015)
Agriculture		−0.207***	−0.203***	−0.203***
		(0.013)	(0.013)	(0.019)
Family size		0.016***	0.015***	0.015***
		(0.004)	(0.004)	(0.005)
Employment status		0.161***	0.160***	0.160***
		(0.011)	(0.011)	(0.012)
Home owner		0.047***	0.044***	0.044**
		(0.013)	(0.013)	(0.017)
Transfer income		−0.002	−0.002	−0.002
		(0.001)	(0.001)	(0.002)
GDP per capita		0.209***	0.110***	0.110***
		(0.013)	(0.017)	(0.023)
Regional fixed effects	NO	NO	YES	YES
Constant	3.417***	1.434***	2.455***	2.455***
	(0.007)	(0.146)	(0.187)	(0.252)
Observation	32,058	32,058	32,058	32,058
	0.049	0.180	0.182	0.182

***p < 0.01, **p < 0.05. Standard errors clustered at the individual level are reported in parentheses in columns (1)–(3), and standard errors clustered at the household level are reported in parentheses in column (4).

### Endogeneity

The results of the Ordinary Least Squares (OLS) estimation in Table 3 may be biased due to problems with endogeneity. First, omitted variable bias may occur if individuals' willingness to use mobile payment is correlated with unobserved factors that affect their physical health. Second, the effect of mobile payment on individuals' physical health may be subject to bias from reverse causality since physical health may also have a strong influence on the use of mobile payment. Third, not controlling for potential measurement errors in mobile payment and physical health may result in an upward or downward bias in the estimated effect of mobile payment on physical health. Thus, potential endogeneity issues need to be considered.

To address the endogeneity problems, we estimate the impact of mobile payment on individuals' physical health using a Two-stage Least Squares (2SLS) strategy. Similar to Bucher-Koenen and Lusardi (29), we employ exposure to mobile payment by other individuals in the same village as an instrumental variable (IV).

To confirm the validity of the IV, several relevant statistical tests have been employed. First, the value of the minimum eigenvalue statistic in the Wald test is 2203.64, which is far above the critical value (8.96), meaning that the null hypothesis that the IV is not valid can be rejected (30). Column (1) of Table 4 reports the results of first stage of the 2SLS, which indicate that the F-value is 565.45, well-above the critical value of 10 (31), and the IV has a positive effect on mobile payment. Furthermore, we employ the Limited Information Maximum Likelihood (LIML) method to study the health impact of mobile payment. The LIML results are consistent with the results of 2SLS in Table 4. These results indicate that the weak instrument problem is not a major concern in our study.

**Table 4:** The impact of mobile payment on physical health—accounting for endogeneity.

**Variable**	**2SLS**		**LIML**
	**First stage**	**Second stage**	**LIML**
Mobile payment		0.435***	0.435***
		(0.064)	(0.064)
IV	0.709***		
	(0.025)		
Control variable	YES	YES	YES
Regional fixed effects	YES	YES	YES
Constant	−0.160	2.569***	2.569***
	(0.148)	(0.257)	(0.257)
Observation	32,058	32,058	32,058
*F*-value	565.45	——	——
	0.255	0.165	0.165

***p < 0.01, **p < 0.05, *p < 0.10. Standard errors clustered at the household level are reported in parentheses.

Column (2) of Table 4 reports the results of the second stage of the 2SLS, which indicate that mobile payment has a positive impact on individuals' health status. Column (3) reports the results of the LIML estimation. The estimated coefficient of the health impact of mobile payment is still positive and significant at the 1% level. These results confirm that mobile payment can be said to affect individuals' physical health even after accounting for endogeneity problems.

### Robustness check

In order to check the robustness of our results, we use various additional tests to examine the impact of mobile payment on health. The first one is to check for self-selection bias by using the Propensity Score Matching (PSM) method. In the second, we replace the health indicator with an alternative measure. Finally, we employ a different methodology to check the robustness of our results.

#### Self-selection bias

Individuals' decision about whether to use mobile payment is not exogenous and random but rather is affected by external factors such as their education level, work, age, marital status, children and so on. As such, there might be an issue with self-selection. To address this potential selection bias, we employ the PSM method to estimate the health impact of mobile payment. The core idea of the PSM method is matching a suitable counterfactual control group (individuals who do not use mobile payment) with the treatment group (individuals who use mobile payment).

First, we estimate a propensity score according to the characteristics of the control variables. Second, depending on the propensity score, we decide the matching partners for each treated individual. Finally, we compute the mean of the difference between control group and treatment group, and we get the average effect of mobile payment on health level (Average Treatment Effect on Treated, ATT). The equation is as follows:


(2) $$\begin{array}{l} \tau_{ATT}\text{=}{\text{E}}_{p\left(x\right)|D=1}\left[E\left(\text{Healt}h_{1}-\text{Healt}h_{0}\right)|D=1,p\left(x\right)\right] \end{array}$$

To compute the PSM estimation:


(3) $$\begin{array}{l} \tau_{ATT}^{PSM}=E_{p(x)|D=1}[E(\text{Healt}h_{1}|D=1,p(X))\\ \;-E(\text{Healt}h_{0}|D=0,p(X))] \end{array}$$

**Figure 1:**
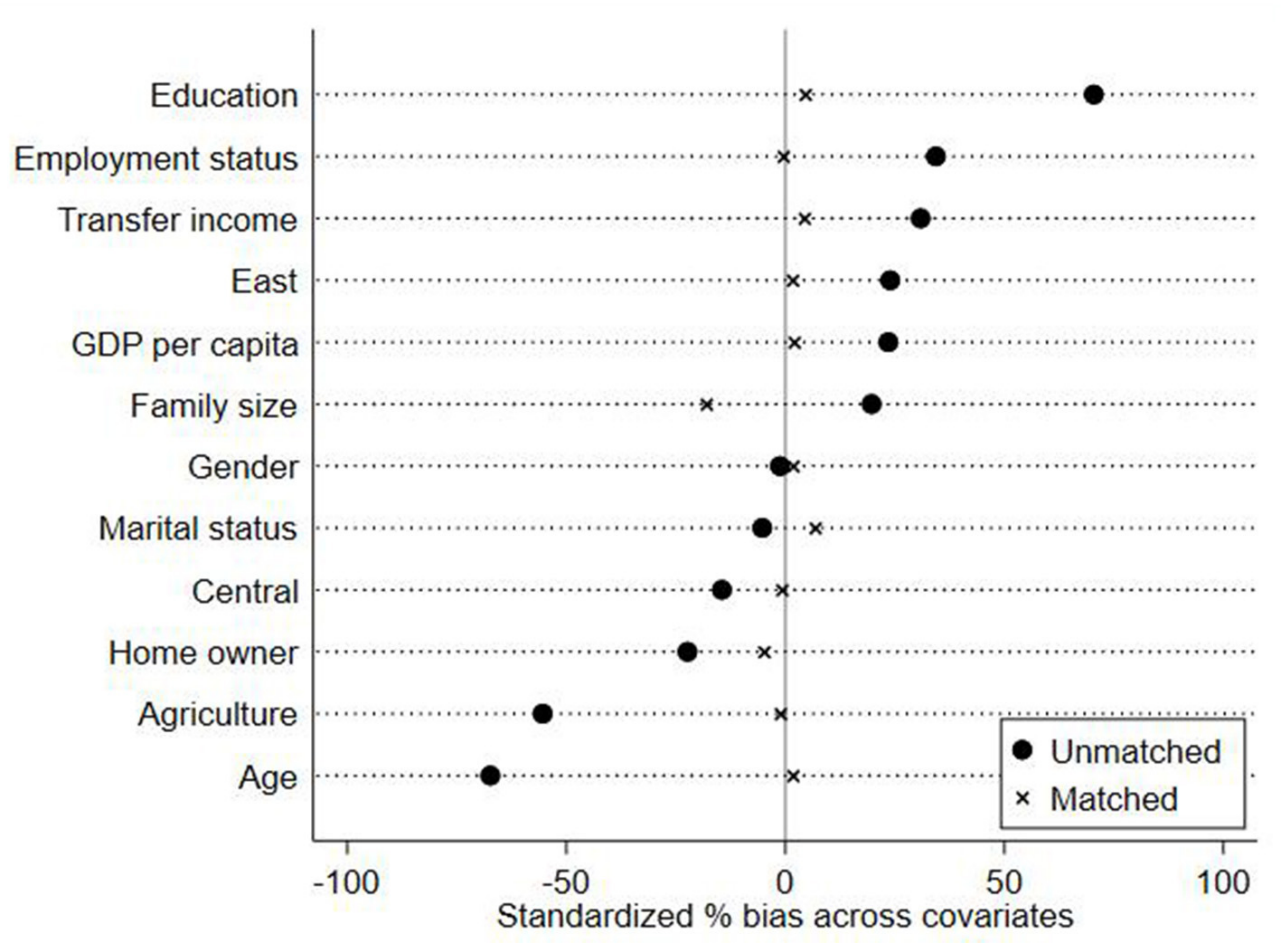
Standardized bias before and after matching.

To apply PSM, the assumption of conditional independence must be satisfied. That is, given a set of observable covariates*p*(*X*), individual health status must be independent of mobile payment. Figure 1 depicts the standardized bias. The figure shows the standardized bias of all variables is <10% after matching (we use k-nearest neighbor matching with k = 4). These results indicate that the covariates are balanced in the two groups.

**Figure 2:**
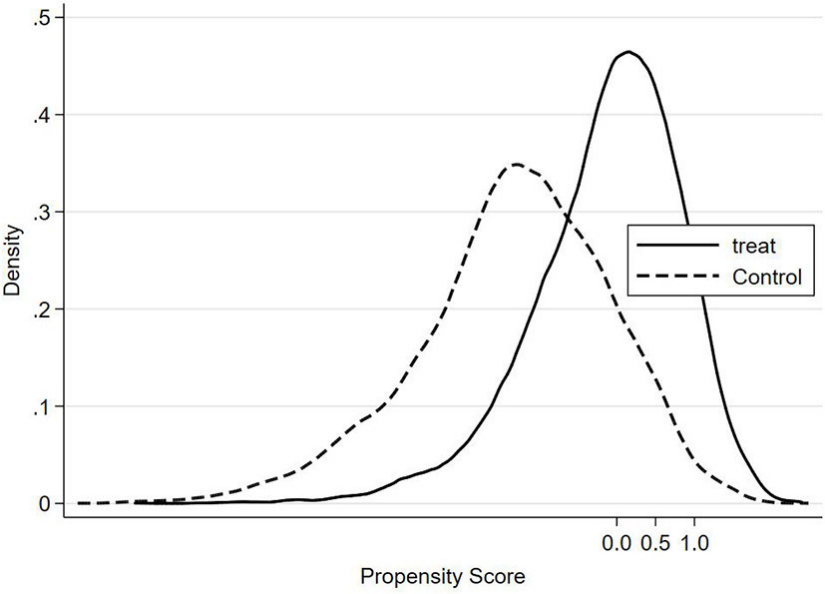
Density distribution of the propensity score (Before matching).

**Figure 3:**
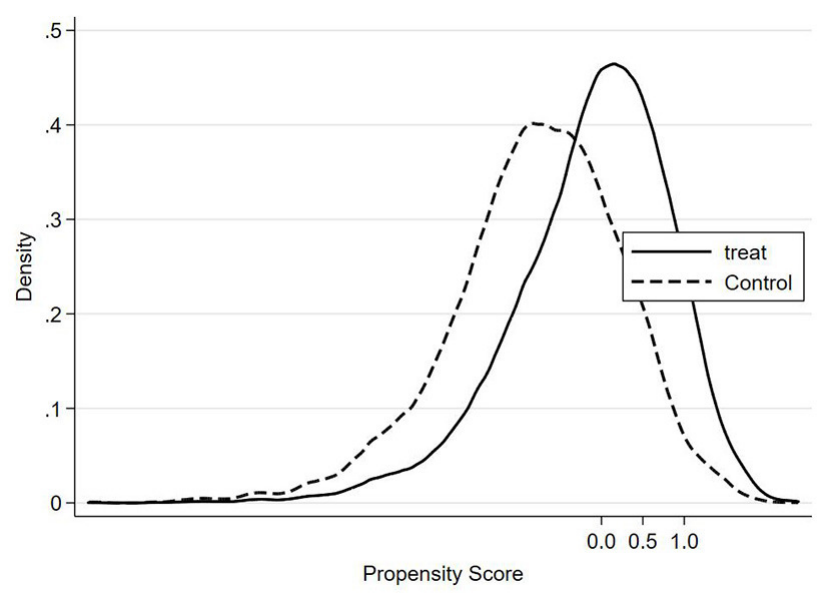
Density distribution of the propensity score (After matching).

In addition to the assumption of conditional independence, the assumption of common support must also be satisfied. The assumption of common support ensures that the propensity score of treatment group and control group are in the same range. Figures 2, 3 show the density distribution of the propensity score before and after matching, respectively. These two figures indicate that only a small number of observations will be lost after matching.

According to Rosenbaum and Rubin (32), there are six matching methods that could be used in this paper. They include k (k = 1, k = 4) nearest-neighbor matching, radius matching, kernel matching, local linear regression matching, and spline matching. The results in Table 5 show that the ATT of these six matching methods is positive and significant, indicating that mobile payment has a positive impact on health.

**Table 5:** The impact of mobile payment on health (accounting for self-selection).

**ATT**	**Nearest neighbor matching (k** = **1)**	**Nearest neighbor matching (k** = **4)**	**Radius matching**	**Kernel matching**	**local linear regression matching**	**spline matching**
Health	0.982***	0.916***	0.991***	1.008***	0.976***	0.991***
	(0.297)	(0.258)	(0.442)	(0.240)	(0.302)	(0.213)

Robust standard errors in parentheses; ***denote significance at the 1% level; Since the results of spline matching coefficients do not report the standard error, the standard errors in column (6) are based on Bootstrap sampling (1,000 times).

#### The replacement of health indicator

In the previous section, we employ the self-evaluation of health registered as a score from 1 to 5 to represent individuals' health status. In order to confirm the reliability of our baseline results, we use another measurement of health. Specifically, we replace the dependent variable with a dummy. This dummy takes the value 1 if the individuals' self-evaluation of their health is “good” or “very good,” and 0 otherwise. As our dependent variable is now a dummy, it allows us to use an IV-Probit model to investigate the impact of the use of mobile payment on health. The results are shown in column (1) of Table 6. The coefficient of mobile payment is positive and statistically significant at the 1% level, indicating that mobile payment has a positive effect on health.

**Table 6:** Results for robustness check.

**Variable**	**IV-probit**	**IV-ordered probit**
Mobile payment	0.476***	0.391***
	(0.067)	(0.039)
Constant	YES	YES
Control variable	YES	YES
Region fixed effects	YES	YES
Observation	32058	32058

***p < 0.01. Standard errors are reported in parentheses.

#### Alternative methodology

The health status score is an ordinal variable: a higher score represents better health status. Following Hassen (33), we employ an IV-Ordered Probit model to analyze the effect of mobile payment on health. Column (2) in Table 6 reports the estimated results, indicating that mobile payment has a positive effect on health.

### Heterogeneity analysis

The results can be driven by different characteristics of individuals in the sample, thus, further analysis of such heterogeneity is conducted in this subsection. We first check the heterogeneity of the effect between rural and urban regions, and then investigate the impact of mobile payment on health across different educational levels.

#### Heterogeneity of the effect between rural and urban regions

Rural regions differ from urban regions in many respects, such as geographic environment, attitudes to consumption, and consumption habits. Therefore, the impact of mobile payment on the health of rural and urban residents may not be homogeneous. To study the heterogeneous effect of mobile payment on health between urban and rural individuals, we split our sample into urban and rural groups and apply the 2SLS technique to each group. The results are shown in columns (1) and (2) of Table 7. They show that the impact of mobile payment is positive and statistically significant for both rural and urban regions; however, the magnitude of the effect is greater for rural individuals than for urban ones. The results for the *p*-value obtained by Bootstrap sampling (1,000 times) confirm that we can reject the null hypothesis (The null hypothesis of no significant difference between rural and urban). This indicates that mobile payment has a greater impact on health for rural individuals than for urban cohorts.

**Table 7:** Heterogeneity analysis.

**Variable**	**Rural**	**Urban**	**High level of education**	**Low level of education**
Mobile payment	0.908***	0.373***	0.142**	0.810***
	(0.220)	(0.066)	(0.070)	(0.099)
Constant	YES	YES	YES	YES
Control variables	YES	YES	YES	YES
Regional fixed effects	YES	YES	YES	YES
Observations	7,536	24,522	14,539	17,519
*P*-value	0.000***		0.000***	

***p < 0.01, **p < 0.05. P-value is used to test the significance of the difference in the coefficient of mobile payment between groups, which is obtained through 1,000 Bootstrap replications.

#### Heterogeneity between education levels

The impact of mobile payment on health can vary according to education level. To examine this heterogeneous effect, we separate the sample into two different groups by years of education: a group of more-educated individuals who have completed more than 10 years of education (the mean value of our observations), and a less-educated group with fewer years. The 2SLS method is again employed to study the effect of mobile payment for these two groups. Columns (3) and (4) of Table 7 report the results, which indicate that the impact of mobile payment is positive and statistically significant for both groups. However, the magnitudes of the two coefficients show that the effect of mobile payment is greater for less-educated individuals. The results of the *p*-value obtained by Bootstrap sampling (1,000 times) lead us to reject the null hypothesis (The null hypothesis of no significant difference between the group of high level of education and low level of education). This indicates that mobile payment has a greater impact on health for less-educated individuals than more-educated ones.

## Mechanism

### Purchase of private health insurance

To examine the possibility that private health insurance might be a channel through which mobile payment has an impact on health, we use two measures of private health insurance as our dependent variable. First, we create a dummy variable for private health insurance: it equals 1 if the individual purchases any private health insurance, and zero otherwise. Second, the integrated purchase level of private health insurance is also considered by using the amount of the insurance. We estimate an IV-Probit model to study the impact of mobile payment on the purchase of private health insurance. Since some people might not have any private health insurance at all and the value would be 0 for these people, we employ an IV-Tobit model to examine the impact of mobile payment on the level of private health insurance purchase. Results are shown in Table 8. The positive coefficient of mobile payment in column (1) indicates that the use of mobile payment has a positive impact on the purchase of private health insurance. Furthermore, the results in column (2) show that the effect of mobile payment on the level of private health insurance purchase is positive and statistically significant. These results suggest that the use of mobile payment might promote the purchase and the amount of private health insurance, ultimately improving individuals' health.

**Table 8:** Mechanism test 1: the impact of mobile payment on private health insurance.

**Variable**	**(1)**	**(2)**
Mobile payment	1.104***	1.008***
	(0.151)	(0.201)
Constant	YES	YES
Control variables	YES	YES
Region fixed effects	YES	YES
Observations	31,727	31,648

***p < 0.01. Standard errors clustered at the household level are reported in parentheses.

### Family leisure consumption

To study the mechanism between mobile payment and individual's physical health, we explore two lines: the total amount of leisure spending and the proportion of spending on leisure activities. The family leisure consumption includes the expenditure on books, magazines, CD, films, bars, Internet cafes, pets, amusement parks, toys, sporting equipment and tourism expenses. We add up the money spent on leisure activities including newspapers, magazines, music, films, bars, web bars, pets, amusement parks, toys, sporting equipment and tourism expenses.

There might be some zero values in our dependent variable because some families might not spend any money at all on leisure activities. Thus, to investigate the effect of mobile payment on the spending on leisure activities, we estimate an IV-Tobit model. The positive results in columns (1) and (2) of Table 9 show that the use of mobile payment has a positive impact on the proportion and amount of leisure spending, indicating that the use of mobile payment has an impact on individual health through the channel of increased spending on leisure activities.

**Table 9:** Mechanism test 2: the impact of mobile payment on family leisure consumption.

**Variable**	**(1)**	**(2)**
Mobile payment	1.186***	0.641***
	(0.074)	(0.074)
Constant	YES	YES
Control variables	YES	YES
Region fixed effects	YES	YES
Observations	30,943	23,138

***p < 0.01. Standard errors clustered at the household level are reported in parentheses.

## Discussion

Given the important role played by mobile payment, some studies try to understand whether mobile payment has an impact on health; to date, however, there is only indirect evidence of a relationship. For instance, mobile payment helps to reduce transaction costs, thereby improving the accessibility of financial services (34). Some mobile payment platforms (such as WeChat, Alipay, etc.) can provide small loans and alleviate individuals' credit constraints, which can effectively increase the family's use of health care services (35). At the meantime, Jack and Suri (36) found that mobile payment can smooth the consumption of food and somewhat reduce the impact of negative shocks such as unemployment or major disease. Another strand of the literature on mobile payment focuses on its possible impact on people's consumption (37) and societal welfare (38, 39). In addition, the factors that influence the use of mobile payment is another area of interest in the existing academic research (40–42). It can be concluded that previous studies have either only explored the indirect impact of mobile payment on individual's health, or examined the factors that influence the use of mobile payment.

Different from existing research, based on the data from 2017 CHFS, we estimate the effect of mobile payment on individual's physical health, providing the direct evidence of a relationship between mobile payment and individual's physical health. In research methodology, most of the previous studies relevant mobile payment do not consider the endogenous problem and selection bias. To accurately identify the impact of mobile payment on individual's physical health, this paper considers the endogeneity and selection bias of mobile payment. To address the endogeneity problems, we employ the 2SLS method to investigate the impact of mobile payment on individual's physical health. Considering the selection bias, we employ the PSM method to estimate the health impact of mobile payment. We found that mobile payment has a positive impact on individual's physical health.

Private health insurance is an essential part of the insurance industry, and it is an important supplement to the national health care system (43, 44). It can reduce individual's health care expenditure, and improve the accessibility of medical services. Furthermore, private health insurance can reduce the psychological burden caused by disease, and improve the willingness to seek medical treatment. A large number of studies have demonstrated that private health insurance has a positive impact on health (23–25). Mobile payment facilitates the purchase of private health insurance and thus reduces transaction costs. For example, WeChat and Alipay provide various kinds of private health insurance online, which reduce the search costs and transportation cost of purchasing private health insurance. The convenient process helps mobile payment users to purchase private health insurance and thus eases access to health services. Eventually, mobile payment might further improve the health of individuals by promoting the purchase of private health insurance. The results of mechanisms prove that the private health insurance is shown to be the potential mechanism through which the impact of mobile payment on individual's physical health.

Furthermore, the results of mechanisms also indicate that mobile payment have a positive impact on household consumption, which are in line with the previous research (36, 37). The results can be explained by the following reasons: the mobile payment platforms may promote some information about leisure for users, and improve the household leisure consumption. Compared to traditional physical cash, mobile payment can make the payment process more convenient (45). It can also reduce the stress of paying and increase the net utility of consumption. Consumption can be considered as an investment in health (46). Previous studies show that entertainment activities have a positive impact on health (45). In this case, the consumption on leisure items such as films, TV, magazines, amusement parks, and travel might have a positive influence on an individual's health. For these reasons, the use of mobile payment can promote the consumption of leisure activities, and further improve individuals' health status.

The limitation of this paper is as follows: first, this study does not include sophisticated theoretical framework to reveal the causal relationship between mobile payment and individual's physical health. Second, the measurement of physical health is based on the respondents' self-evaluation of health, it is very general measurement of individual's physical health. Third, individual's mental health might be related to mobile payment. Given the lack of the relevant data to mental health, we do not estimate the effect of mobile payment on individual's mental health. Finally, we investigate the short-run effects of mobile payment on individual's physical health. Regretfully, due to data constraints, we fail to consider the long-term impact of mobile payment. An interesting future research avenue could be projected on the long-term and dynamic effects of mobile payment on individual's physical health and mental health.

## Conclusions and policy implications

In this paper, we investigate the causal relationship between mobile payment and individuals' physical health using CHFS survey data from 2017. One of the most important findings to emerge from our research is that mobile payment has a positive impact on individuals' physical health. The heterogeneity analyses show that the influence of mobile payment is slightly weaker for urban residents than for rural residents, and is greater for less-educated people than for their better-educated counterparts. Our study also investigates the mechanisms through which mobile payment can have an impact on physical health, indicating that mobile payment influences physical health through its effect on the purchase of private health insurance and spending on family enjoyment.

Through the research in this article, we can draw a number of policy implications. First, against the background of the Covid-19 pandemic, which is still a threat around the world, individuals use mobile payment tools more often to carry out contactless routine transactions. By so doing, consumers can avoid direct exposure to the coronavirus, thereby reducing the possibility of contracting the virus and protecting their health. Second, Private investors related to the health industry could harness the convenience of mobile payment by making more cooperative connections with payment platforms to promote health services or by offering discounts. With the help of modern network and information technology, investors will benefit the symmetric information in the market and provide their services in low marginal costs. For consumers, it will receive the services in a lower price and increases the flexibility in product selection. Finally, the government should combine the internet, big data, cloud computing, artificial intelligence, blockchain and other technologies to vigorously support innovation by third-party payment companies and encourage them to enrich the mobile payment market and enhance the value of mobile payment. In addition, while guiding financial institutions to provide financial support for health care, the government should also strengthen the risk management of third-party payment companies in order to reduce their security risks and improve consumer protection.

## Data availability statement

The raw data supporting the conclusions of this article will be made available by the authors, without undue reservation.

## Author contributions

Conceptualization: RZ, YZ, and JX. Methodology, software, resources, and visualization: RZ. Validation, data curation, writing—original draft preparation, supervision, and project administration: RZ and YZ. Formal analysis: RZ and JX. Investigation, writing—review and editing, and funding acquisition: YZ. All authors contributed to the article and approved the submitted version.

## Funding

This research was funded by the National Social Science Foundation Youth Project (20CFX054).

## Conflict of interest

The authors declare that the research was conducted in the absence of any commercial or financial relationships that could be construed as a potential conflict of interest.

## Publisher's note

All claims expressed in this article are solely those of the authors and do not necessarily represent those of their affiliated organizations, or those of the publisher, the editors and the reviewers. Any product that may be evaluated in this article, or claim that may be made by its manufacturer, is not guaranteed or endorsed by the publisher.
